# Supplementary material for: Importance of the Cysteine-Rich Domain of Snake Venom Prothrombin Activators: Insights Gained from Synthetic Neutralizing Antibodies
Source: Toxins (Basel). 2024 Aug 15;16(8):361. doi: 10.3390/toxins16080361 (PMC11360484; doi:10.3390/toxins16080361)
Supplement: Supplementary file 1 [file toxins-16-00361-s001.zip › toxins-3127782-SI.pdf]

Supplementary information for:

Importance of the Cysteine-Rich Domain of Snake Venom Prothrombin Activators: Insights Gained from Synthetic Neutralizing Antibodies

The PDF file includes:

Figures S1 to S15

Tables S1 to S2

Supplementary Figures

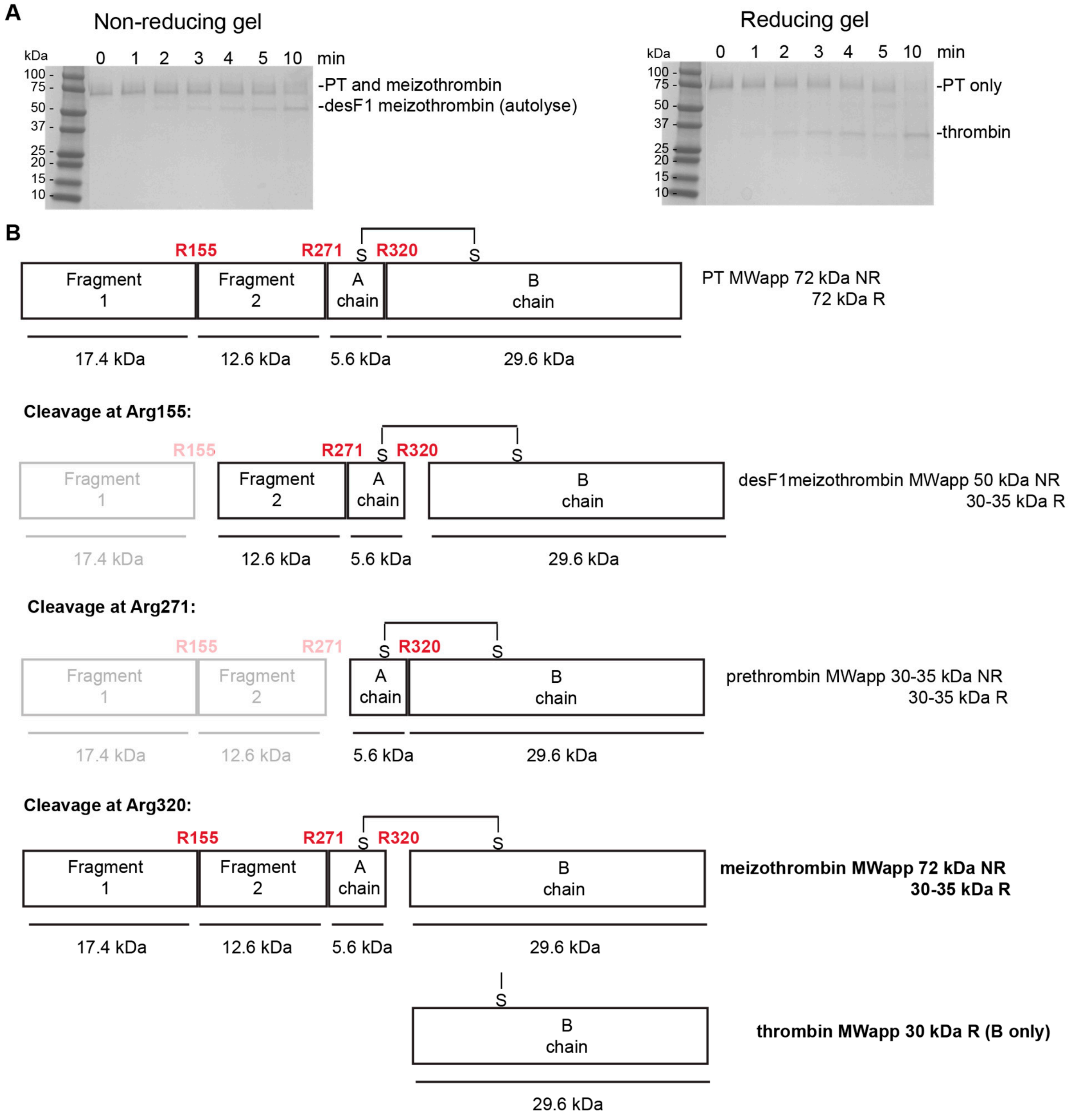

Figure S 1 Meizothrombin formation catalyzed by ecarin. **A)** Reaction conditions: PBS pH 7.4, 22 °C, rEcarin 50 nM, recombinant human prothrombin 1 µM. Aliquots at 0, 1, 2, 3 ,4, 5 and 10 min were quenched with non-reducing or reducing SDS loading dye. Samples were boiled for 5 min and loaded on a gel (150 V, 45 min). **B)** Apparent molecular weights (MWapp) of PT fragments if cleaved at Arg155 (desF1meizothrombin), Arg271 (prothrombin) or Arg320 (meizothrombin and thrombin) in non-reducing (NR) or reducing (R) conditions.

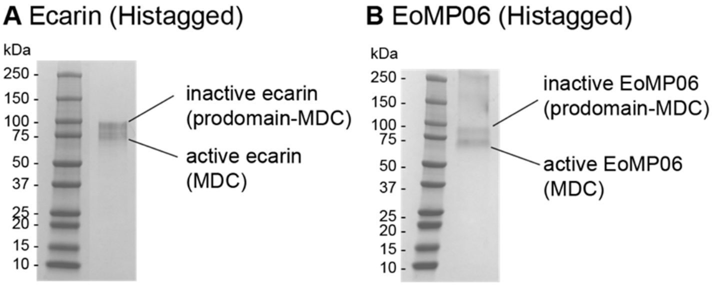

Figure S 2 SDS PAGE of rSVMPs. A) His_{10}-tagged rEcarin. The inactive and active forms of ecarin (with and without prodomain, respectively) can be distinguished. **B)** His_{10}-tagged rEoMP06 with complete pro-domain. The inactive and active forms of EoMP06 (with and without pro-domain, respectively) can be distinguished.

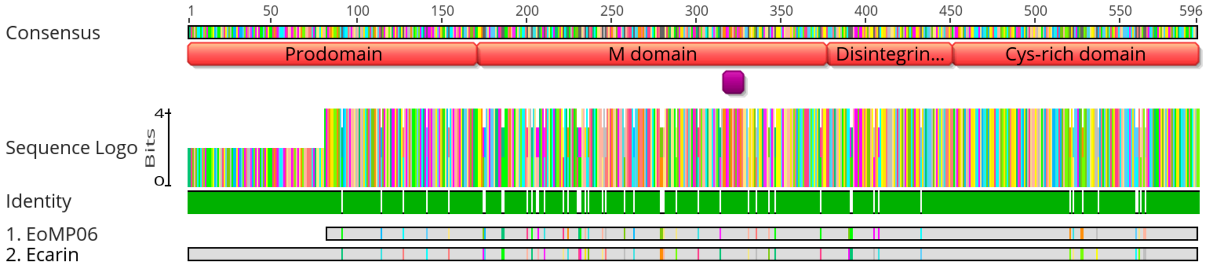

Figure S 3 Sequence alignment of EoMP06 (Uniprot Q6X1T6) with ecarin (Uniprot Q90495) showing incomplete sequence for EoMP06 prodomain. The pro-, M, D-like and C domains are indicated by the red boxes. The conserved HEXXHXXGXXHD motif of the catalytic domain is indicated by the purple box.

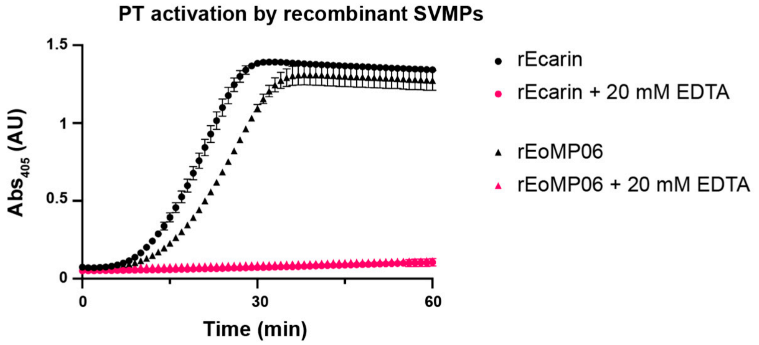

Figure S 4 Human prothrombin (PT) activation by rSVMPs. Reaction conditions: PBS pH 7.4, 37 °C, rEcarin or rEoMP06 2 nM, recombinant human PT 0.2 μM, S-2298 0.5 mM. Reactions done in triplicate and error bars represent the standard deviation on the measurements.

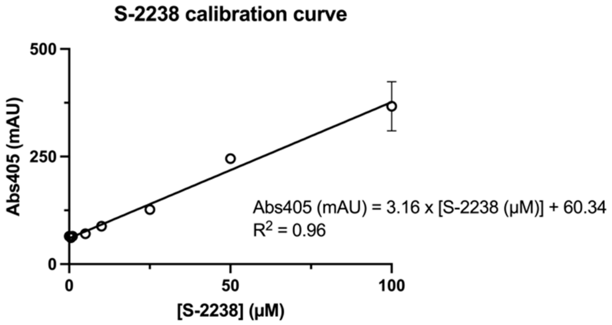

Figure S 5 S-2238 calibration curve to determine para-nitroaniline (pNa) formation. Reaction conditions: 37 °C, PBS, rEcarin 20 nM, recombinant human PT 0.1 µM, S-2238 0.5-100 µM. Reactions were run in triplicate and plateaued absorbance values at 405 nM are indicated (when all S-2238 is converted to pNa).

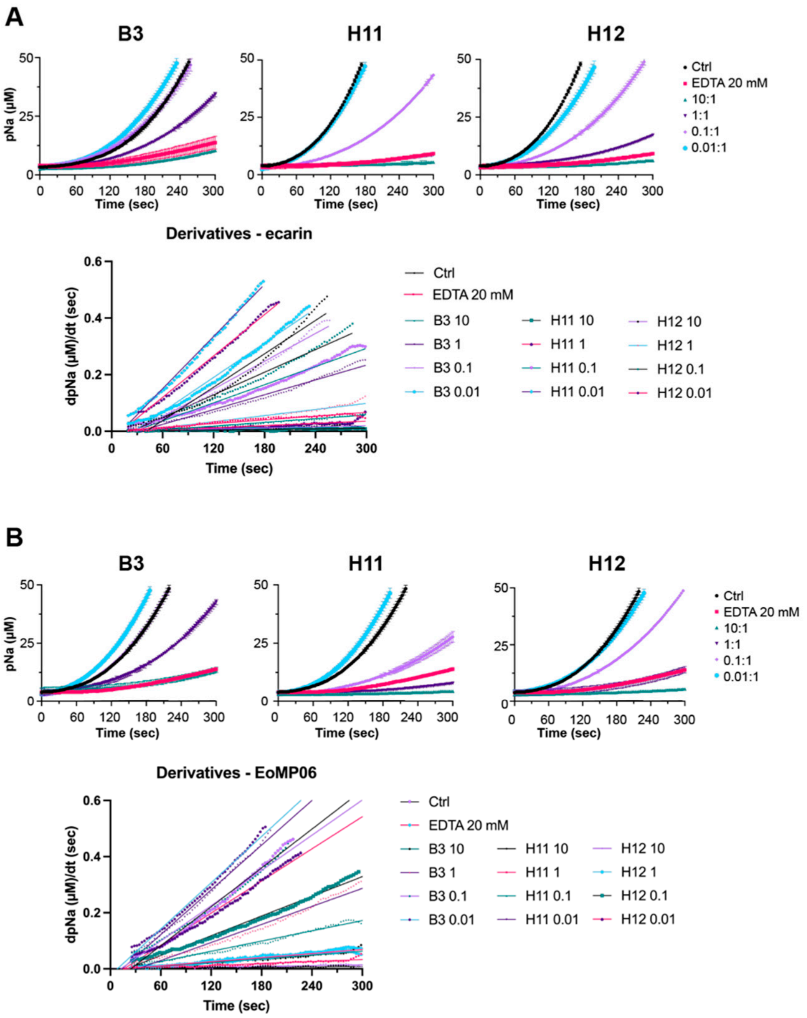

Figure S 6 rEcarin (A) and rEoMP06 (B) rate calculations. Reaction conditions: 37 °C, PBS, rEcarin (A) or rEoMP06 (B) 50 nM, recombinant human PT 0.2 µM, S-2238 0.5 mM, antibodies 0.01 to 10 equivalents of rSVMPs (0.5-500 nM). The concentrations of pNa formed calculated from the calibration curve in Figure S5 are plotted versus time (top graphs). The first derivatives dpNa/dt were then calculated (bottom graphs), and the slopes (second derivatives: d(dpNa/dt)/dt) indicate the rate of PT activation catalyzed by rSVMPs in the presence of different concentrations of B3, H11 and H12 antibodies plotted in Figure 2.

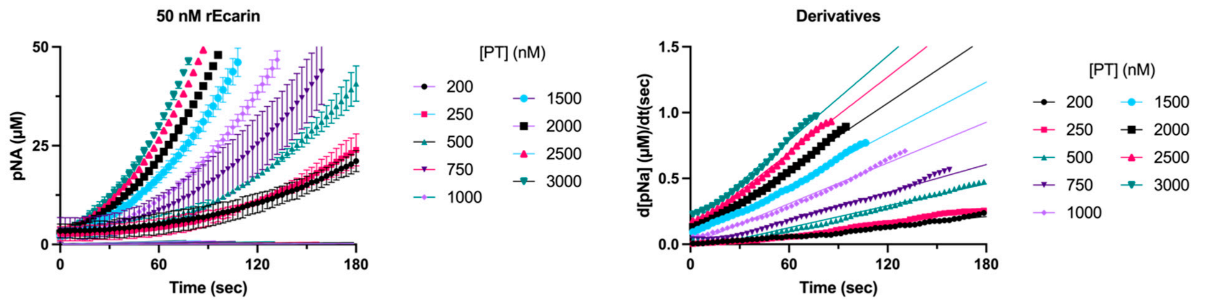

Figure S 7 Rates of PT activation by rEcarin at different concentrations of PT. Reaction conditions: 37 °C, PBS, rEcarin 50 nM, recombinant human PT 0.2-5 μM, S-2238 0.5 mM. **Left:** the concentrations of pNa formed calculated from the calibration curve in Figure S5 are plotted versus time. **Right:** the first derivatives dpNa/dt were then calculated, and the slopes (second derivatives: d(dpNa/dt)/dt) indicate the rate of PT activation catalyzed by rEcarin plotted in Figure 3.

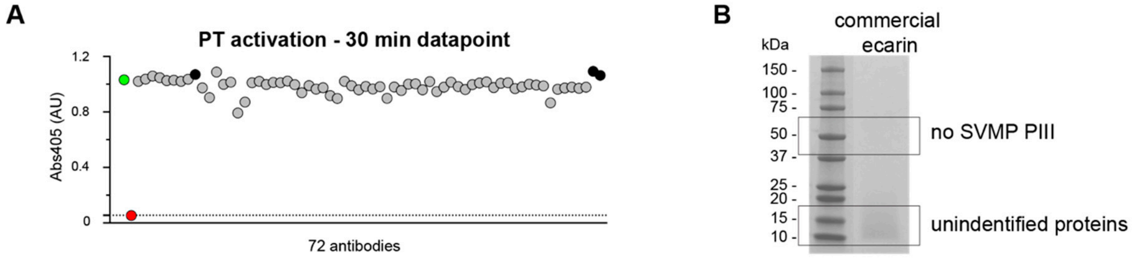

Figure S 8 Commercial ecarin characterization. A) PT activation by commercially available ecarin. Reaction conditions: PBS pH 7.4, 37 °C, commercial ecarin 0.1 µg, recombinant human PT 0.2 µM, S-2238 0.5 mM, antibody 0.1 mg/mL (0.67 µM). The Abs_{405} after 30 min reaction time is depicted. A positive (green dot) and negative (red dot) controls were performed in the absence of antibody, and in the presence of 20 mM EDTA, respectively. B3, H11 and H12 are depicted with black dots. **B)** SDS PAGE gel of commercially available ecarin. No SVMP PIII bands (>50 kDa) are present. The bands observed in the 10-15 kDa range might correspond to C-type lectin-like proteins, but further characterization is needed to confidently assess the nature of these toxins.

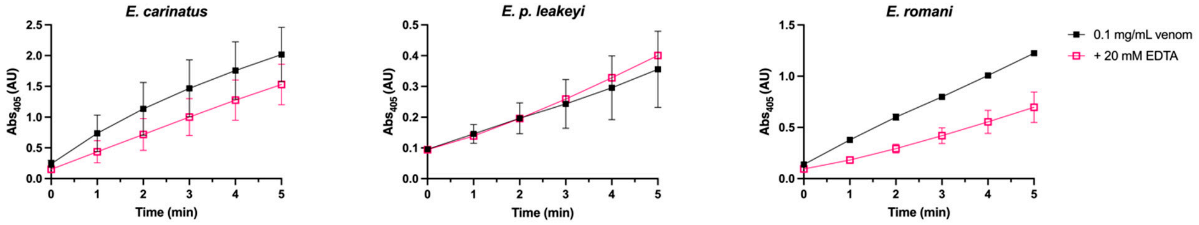

Figure S 9 S-2238 cleavage by whole *Echis* venom. Reaction conditions: 37 °C, PBS, 0.1 mg/mL *Echis* venom (*E. carinatus*, *E. p leakeyi* or *E. romani*), S-2238 0.5 mM, EDTA 0 (black dots) or 20 (red dots) mM. Reactions done in duplicate and error bars represent the standard deviation on the measurements.

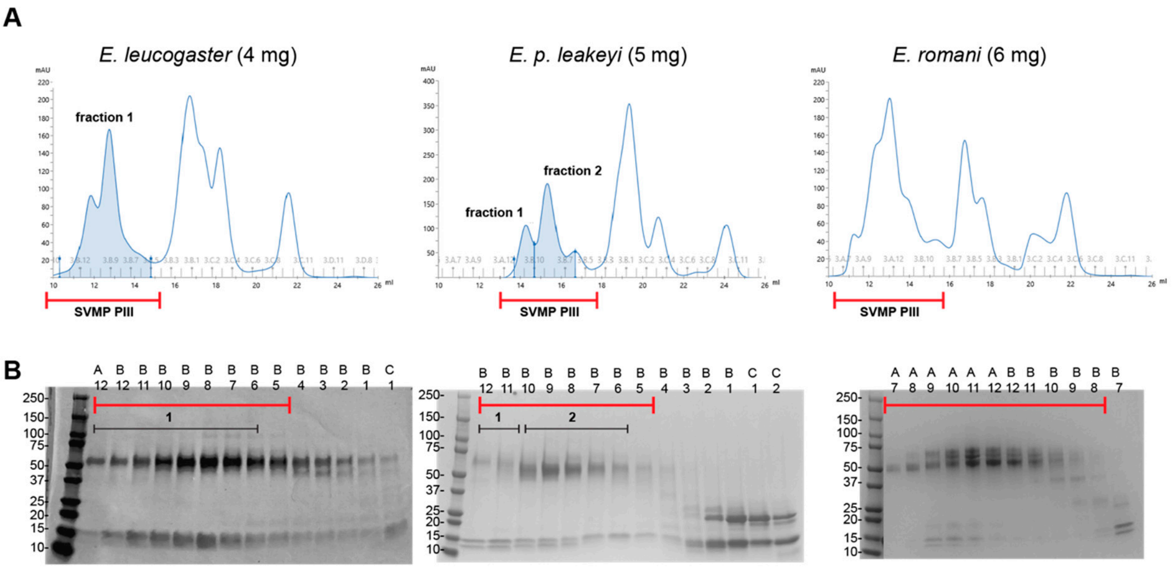

Figure S 10 Purification of SVMP PIII fractions from *Echis* venoms. A) SEC chromatograms depicting the SVMPIII-containing fractions of *E. leucogaster*, *E. p. leakeyi* and *E. romani*. Method: 4-6 mg of lyophilized venoms resuspend in PBS were injected on a Superdex 200 Increase 10/300 GL column. Venom components are separated with an isocratic gradient of PBS at 4 °C at a flowrate of 0.5 mL/min. **B)** Corresponding SDS PAGE gels for SVMP PIII-containing fractions. One and two potential SVMP PIIIs were isolated for *E. leucogaster* and *E. p. leakeyi*, respectively. SVMP PIII-containing fraction of *E. romani* venom precipitated shortly after SEC purification and could therefore not be used for further characterization.

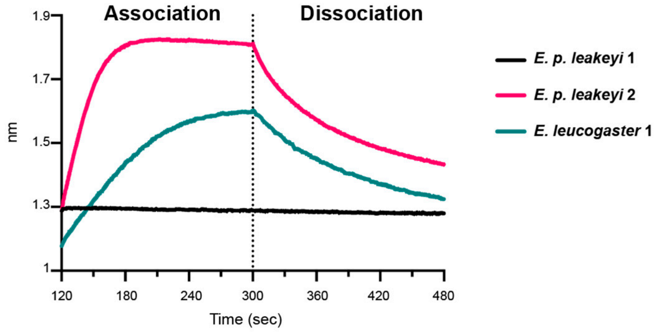

Figure S 11 Binding of isolated SVMP PIIIs from *E. p. leakeyi* and *E. leucogaster* to H11. Bio-Layer Interferometry (BLI) curves showing binding of 1 μM putative SVMP PIII (*E. p. leakeyi* fraction 2 and E. leucogaster fraction 1) to H11.

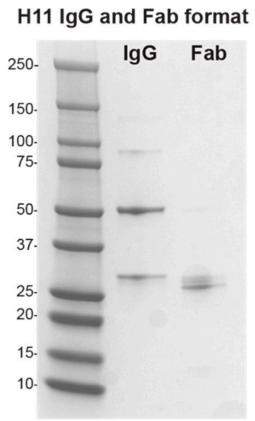

Figure S 7 SDS PAGE analysis of H11 as an IgG and Fab after papain digestion. Under reducing conditions, only 1 band at ~ 25 kDa is present, which corresponds to the reduced H11 Fab.

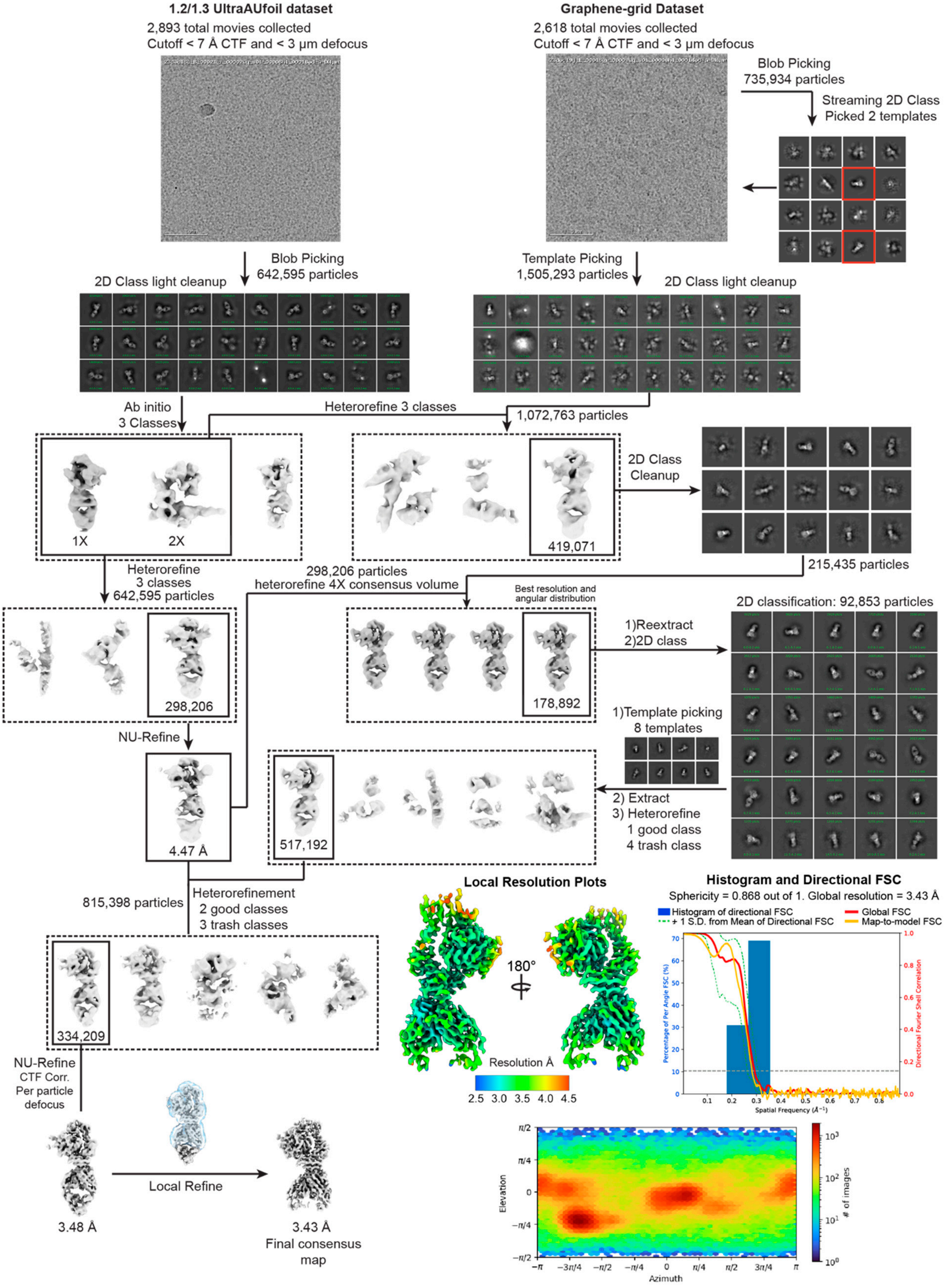

Figure S 8 Cryo-EM processing workflow

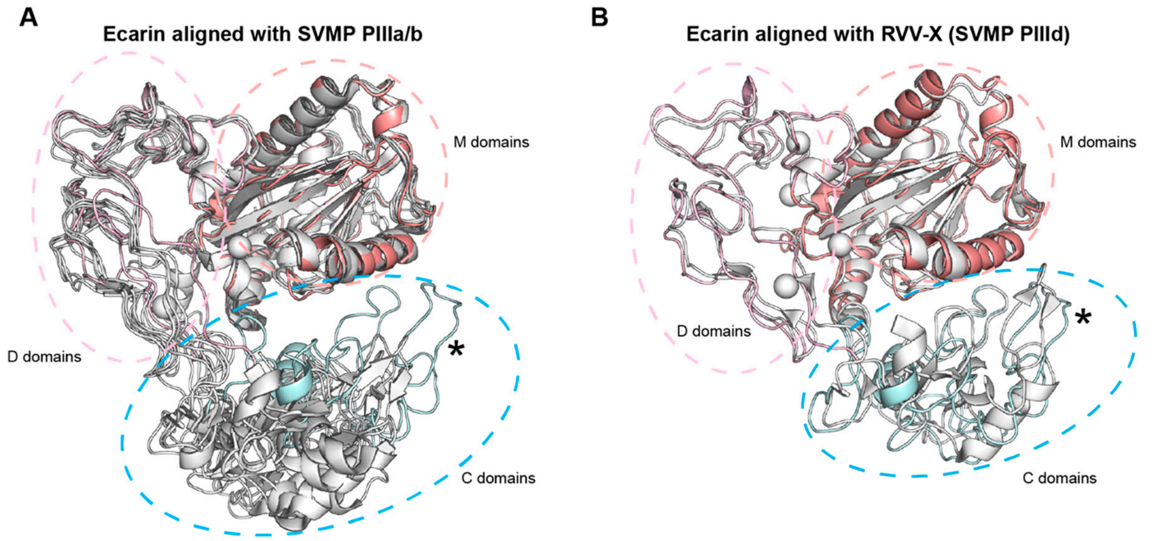

Figure S 9 Comparison of ecarin with SVMPIII crystal structures. A Ecarin aligned with SVMP PIII-a/b: Vap2 (PDB 2DW0), AaHIV (PDB 3HDB), atragin (PDB 3K7L) and bothropasin (PDB 3DSL). The RMSD calculated for the MDC domains range from 4.6 to 5.8 Å. **B** Ecarin aligned with SVMP PIII-d RVV-X without lectin proteins (PDB 2E3X). RMSD = 1.8 Å for the MDC domains. Ecarin M, D and C domains are depicted in salmon, pink and pale cyan, respectively. The other SVMP PIIIs are depicted in white. Ecarin C domain loops interacting with M domain are shown with a "*" for clarity. The structures are aligned on ecarin M domain.

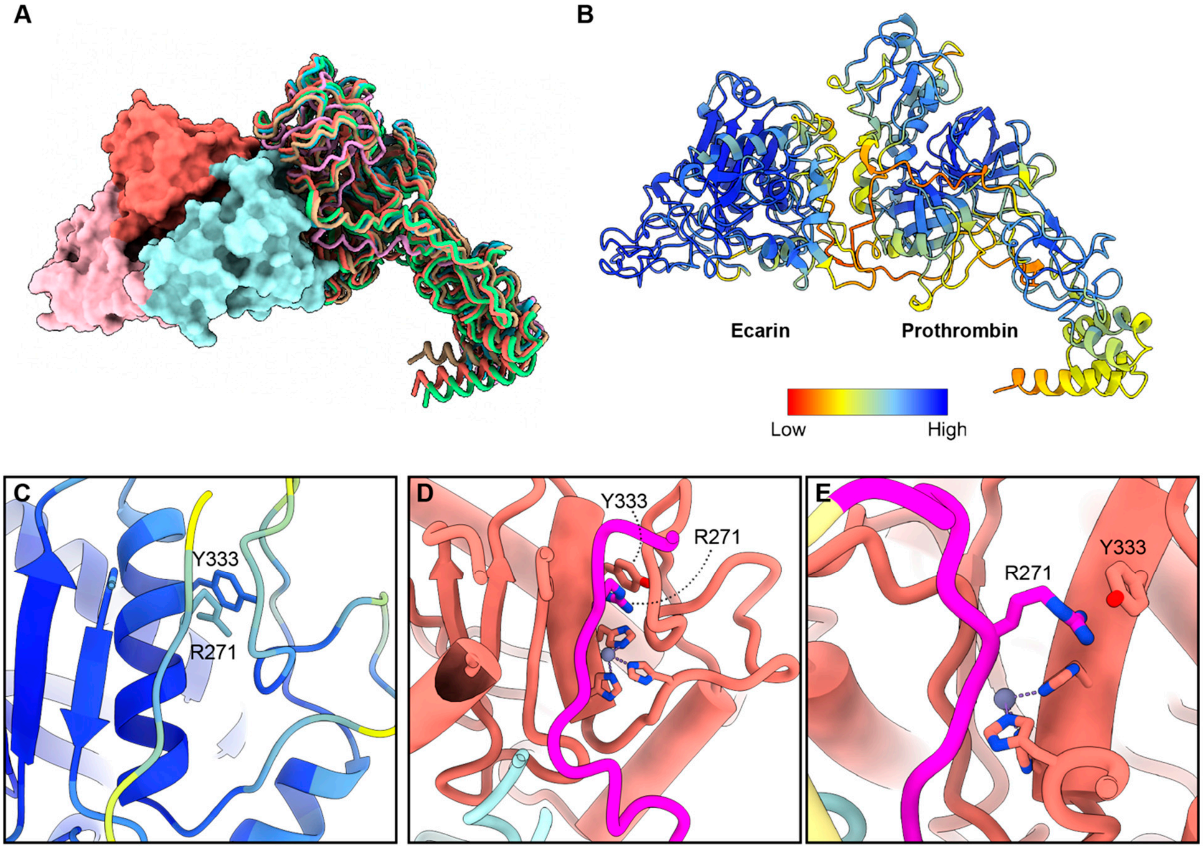

Figure S 10 AlphaFold 2 predicted model of ecarin–PT complex. A) Overlay of five AlphaFold 2.0 generated ecarin-PT complexes. Ecarin is rendered as surface with M domain salmon, D domain pink, and C domain cyan. **B)** pLDDT score mapped onto AlphaFold generated complex. **C)** pLDDT score mapped onto the secondary PT cleavage site in the active site of ecarin. **D, E)** Two views of the proposed coordination of the secondary PT cleavage site (magenta) positioned in the ecarin active site.

Supplementary Tables

Table S1 Binding kinetics of B3, H11 and H12 obtained with an Octet HTX system. K_D: equilibrium dissociation constant; ka: association rate constant; kdis: dissociation rate constant.

Antibody	[ecarin] (nM)	K_D (M)	ka (1/Ms)	ka Error	kdis (1/s)	kdis Error	Assoc R^2
**B3**	1000	**5.332E-07**	9.775E04	3.574E02	5.213E-02	1.062E-04	0.9988
	500	**4.503E-07**	1.109E05	5.369E02	4.992E-02	8.521E-05	0.9987
	250	**4.523E-07**	1.049E05	1.296E03	4.746E-02	8.164E-05	0.9953
	125	**5.196E-07**	8.891E04	2.713E03	4.620E-02	1.050E-04	0.9928
**H11**	500	**9.914E-08**	2.337E05	2.576E03	2.317E-02	1.384E-04	0.9740
	250	**8.936E-08**	2.329E05	1.708E03	2.081E-02	1.127E-04	0.9938
	125	**7.941E-08**	2.331E05	1.573E03	1.851E-02	8.470E-05	0.9979
	62.5	**7.994E-08**	1.987E05	1.708E03	1.589E-02	5.886E-05	0.9991
**H12**	500	**1.656E-07**	2.024E05	1.333E03	3.351E-02	1.791E-04	0.9947
	250	**1.478E-07**	1.973E05	8.690E02	2.916E-02	1.352E-04	0.9995
	125	**1.328E-07**	1.976E05	1.162E03	2.624E-02	9.550E-05	0.9996
	62.5	**1.498E-07**	1.586E05	1.697E03	2.375E-02	5.612E-05	0.9992

Table S2 Cryo-EM data collection, refinement, and validation

	**Ecarin-H11 Complex**<br>**EMDB: EMD-45728**<br>**PDB: 9CLP**
Microscope	Talos Arctica
Voltage (keV)	200
Detector	K2 (counting)
Magnification (nominal/calibrated)	73,000X / 88,339X
Exposure navigation	Image shift to 49 holes
Data acquisition software	Leginon
Total electron exposure ($e^-/Å^2$)	54
Exposure rate (e^-/pixel/sec)	3.46
Frame length (ms)<br>Number of frames per micrograph	200<br>25
Pixel size (Å)	0.566
Nominal defocus range (μm)	0.9-1.6
Measured defocus range (μm)	0.46-3.0
Micrographs collected (no.)	5,782
**Reconstruction**	
Image processing package	CryoSparc
Total extracted particles (no.)	2,147,888
Refined particles (no.)	334,209
Final particles (no.)	334,209
Symmetry imposed	C1
Resolution (Å)	
FSC 0.5 (unmasked / masked)	4.49 / 3.89
FSC 0.143 (unmasked / masked)	4.04 / 3.41
Resolution range (local)	2.891 – 10.551
3DFSC Sphericity	0.868
Sharpening B-factor ($Å^2$)	-150
**Model Composition**	
Protein residues	657
Ligands	4
**Model Refinement**	
Refinement package	Phenix
CC (volume / mask)	0.75 / 0.77
B-factors ($Å^2$)	
Protein	76.18
Ligand	83.05
R.m.s. deviations	
Bond lengths	0.002
Bond angles (°)	0.521
**Validation**	
Map-to-model FSC 0.5	3.70
Ramachandran (%)	
Outliers	0
Allowed	2.16
Favored	97.84
MolProbity score	1.29
Poor rotamers (%)	0.00
Clashscore (all atoms)	4.86
C-beta deviations	0.00
CaBLAM Outliers (%)	1.87
EMRinger Score	3.16
Qscore	0.4790
